# Redetermination of the salt hexa­methyl­ene­tetra­minium fumarate

**DOI:** 10.1107/S1600536810052700

**Published:** 2010-12-24

**Authors:** Andreas Lemmerer

**Affiliations:** a Molecular Sciences Institute, School of Chemistry, University of the Witwatersrand, Private Bag 3, PO WITS, 2050 Johannesburg, South Africa

## Abstract

The crystal structure of the title compound [systematic name: 3,5,7-triaza-1-azoniatricyclo­[3.3.1.1^3,7^]decane (*E*)-3-carb­oxy­prop-2-enoate], C_6_H_13_N_4_^+^·C_4_H_3_O_4_^−^, had been determined previously by Bowes *et al.* [Acta Cryst. (2003), B**59**, 100–117]. Their structure contained an approximately 3:1 ratio of fumarate and succinate monoanions disordered over the same position. The succinate anion component forms a similar structural role to the fumarate anion and came about due to an impurity in the starting material, fumaric acid. In this work, the crystal structure of the pure salt is presented, which is identical, apart from the lack of disorder of the anions, to the previous structure. In the crystal, the ions assemble in the solid state, forming chains *via* N^+^—H⋯O^−^ and O—H⋯O^−^ hydrogen bonds, which are linked into a three-dimensional network by C—H⋯O inter­actions.

## Related literature

For the previous synthesis and structure determination, see: Bowes *et al.* (2003 ▶). For graph-set nomenclature of hydrogen bonds, see: Bernstein *et al.* (1995 ▶).
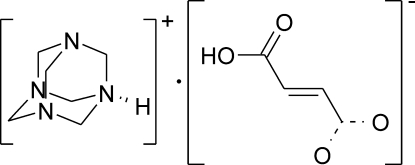


## Experimental

### 

#### Crystal data

C_6_H_13_N_4_^+^·C_4_H_3_O_4_^−^
*M* *_r_* = 256.27
Monoclinic, 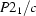

*a* = 6.3020 (3) Å
*b* = 16.0828 (8) Å
*c* = 11.2205 (6) Å
β = 95.930 (2)°
*V* = 1131.15 (10) Å^3^
*Z* = 4
Mo *K*α radiation
μ = 0.12 mm^−1^
*T* = 173 K
0.4 × 0.26 × 0.1 mm

#### Data collection

Bruker APEXII CCD area-detector diffractometer
Absorption correction: integration (*XPREP*; Bruker, 2004 ▶) *T*_min_ = 0.936, *T*_max_ = 0.989
12032 measured reflections
2733 independent reflections
2263 reflections with *I* > 2σ(*I*)
*R*_int_ = 0.114

#### Refinement

*R*[*F* ^2^ > 2σ(*F* ^2^)] = 0.045
*wR*(*F* ^2^) = 0.128
*S* = 1.04
2733 reflections
169 parameters
H atoms treated by a mixture of independent and constrained refinement
Δρ_max_ = 0.36 e Å^−3^
Δρ_min_ = −0.26 e Å^−3^

### 

Data collection: *APEX2* (Bruker, 2005 ▶); cell refinement: *SAINT-Plus* (Bruker, 2004 ▶); data reduction: *SAINT-Plus* and *XPREP* (Bruker, 2004 ▶); program(s) used to solve structure: *SHELXS97* (Sheldrick, 2008 ▶); program(s) used to refine structure: *SHELXL97* (Sheldrick, 2008 ▶); molecular graphics: *ORTEP-3 for Windows* (Farrugia, 1997 ▶) and *DIAMOND* (Brandenburg, 1999 ▶); software used to prepare material for publication: *WinGX* (Farrugia, 1999 ▶) and *PLATON* (Spek, 2009 ▶).

## Supplementary Material

Crystal structure: contains datablocks global, I. DOI: 10.1107/S1600536810052700/bt5439sup1.cif

Structure factors: contains datablocks I. DOI: 10.1107/S1600536810052700/bt5439Isup2.hkl

Additional supplementary materials:  crystallographic information; 3D view; checkCIF report

## Figures and Tables

**Table 1:** Hydrogen-bond geometry (Å, °)

*D*—H⋯*A*	*D*—H	H⋯*A*	*D*⋯*A*	*D*—H⋯*A*
N1—H1⋯O1	0.845 (19)	2.094 (19)	2.8704 (14)	153 (2)
O3—H3⋯O1^i^	1.034 (18)	1.458 (18)	2.4877 (13)	174 (2)
C1—H1*A*⋯O3^ii^	0.99	2.46	3.2708 (16)	138
C3—H3*B*⋯O4^iii^	0.99	2.55	3.4629 (18)	154
C4—H4*B*⋯O4^iv^	0.99	2.48	3.3668 (17)	149
C6—H6*A*⋯O4^iv^	0.99	2.43	3.3352 (18)	152

Symmetry codes: (i) 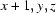; (ii) 
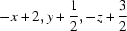
; (iii) 
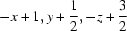
; (iv) 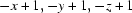.

**Fig. 1.**
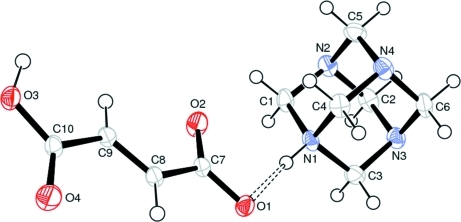
View of (I) (50% probability displacement ellipsoids)

**Fig. 2.**
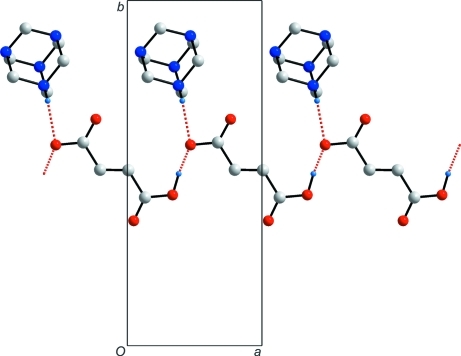
Hydrogen bonding chain of (I) using N+—H···O- and O—H···O- hydrogen bonds (red dashed lines), generated by translation along the a axis.

**Fig. 3.**
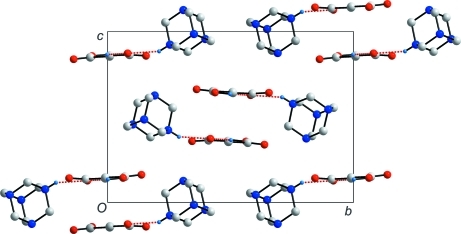
Packing diagram of the four C(7) chains in the unit cell.

## supplementary crystallographic information

### Comment

The fumarate anions forms a C(7) chain (Graph Set notation; Bernstein *et al.*, 1995) along the *a* axis by unit cell translations only. The chain is formed by O—H···O^-^ hydrogen bonds. The hexamethylenetramanium cation is pendant to the chains, and linked to them by N^+^—H···O^-^ hydrogen bonds (Fig. 2). Fig. 3 shows the relative packing of the chains down the *a* axis.

### Experimental

Crystals where grown by slow evaporation at ambient conditions of a methanol solution containing a stoichiometric quantity of hexamethylenetetramine and fumaric acid.

### Refinement

The C-bound H atoms were geometrically placed (C—H bond lengths of 0.95 (ethylene CH) and 0.99 (CH_2_) Å) and refined as riding with *U*_iso_(H) = 1.2*U*_eq_(C). The O-bound H atom and N-bound H were located in the difference map and their coordinates refined freely, with *U*_iso_(H) = 1.5*U*_eq_(O) or 1.5*U*_eq_(N).

### Crystal data

**Table:**

C_6_H_13_N_4_^+^·C_4_H_3_O_4_^−^	*F*(000) = 544
*M**_r_* = 256.27	*D*_x_ = 1.505 Mg m^−^^3^
Monoclinic, *P*2_1_/*c*	Mo *K*α radiation, λ = 0.71073 Å
Hall symbol: -P 2ybc	Cell parameters from 4463 reflections
*a* = 6.3020 (3) Å	θ = 2.2–28.3°
*b* = 16.0828 (8) Å	µ = 0.12 mm^−^^1^
*c* = 11.2205 (6) Å	*T* = 173 K
β = 95.930 (2)°	Block, colourless
*V* = 1131.15 (10) Å^3^	0.4 × 0.26 × 0.1 mm
*Z* = 4	

### Data collection

**Table:**

Bruker APEXII CCD area-detector diffractometer	2263 reflections with *I* > 2σ(*I*)
ω scans	*R*_int_ = 0.114
Absorption correction: integration (*XPREP*; Bruker, 2004)	θ_max_ = 28.0°, θ_min_ = 2.2°
*T*_min_ = 0.936, *T*_max_ = 0.989	*h* = −8→8
12032 measured reflections	*k* = −21→21
2733 independent reflections	*l* = −11→14

### Refinement

**Table:**

Refinement on *F*^2^	0 restraints
Least-squares matrix: full	H atoms treated by a mixture of independent and constrained refinement
*R*[*F*^2^ > 2σ(*F*^2^)] = 0.045	*w* = 1/[σ^2^(*F*_o_^2^) + (0.0679*P*)^2^ + 0.1146*P*] where *P* = (*F*_o_^2^ + 2*F*_c_^2^)/3
*wR*(*F*^2^) = 0.128	(Δ/σ)_max_ = 0.001
*S* = 1.04	Δρ_max_ = 0.36 e Å^−^^3^
2733 reflections	Δρ_min_ = −0.26 e Å^−^^3^
169 parameters	

### Special details

**Table:**

Experimental. Numerical integration absorption corrections based on indexed crystal faces were applied using the *XPREP* routine (Bruker, 2004)
Geometry. All e.s.d.'s (except the e.s.d. in the dihedral angle between two l.s. planes) are estimated using the full covariance matrix. The cell e.s.d.'s are taken into account individually in the estimation of e.s.d.'s in distances, angles and torsion angles; correlations between e.s.d.'s in cell parameters are only used when they are defined by crystal symmetry. An approximate (isotropic) treatment of cell e.s.d.'s is used for estimating e.s.d.'s involving l.s. planes.

### Fractional atomic coordinates and isotropic or equivalent isotropic displacement parameters (Å^2^)

**Table:**

	*x*	*y*	*z*	*U*_iso_*/*U*_eq_	
C1	0.5255 (2)	0.82554 (8)	0.61723 (12)	0.0247 (3)	
H1A	0.5118	0.8385	0.7024	0.03*	
H1B	0.6756	0.81	0.6098	0.03*	
C2	0.2451 (2)	0.91915 (8)	0.55450 (13)	0.0272 (3)	
H2A	0.207	0.9693	0.5057	0.033*	
H2B	0.2281	0.9326	0.6391	0.033*	
C3	0.1515 (2)	0.77886 (9)	0.58785 (13)	0.0262 (3)	
H3A	0.0546	0.7325	0.5615	0.031*	
H3B	0.1334	0.7915	0.6726	0.031*	
C4	0.4040 (2)	0.73544 (8)	0.44620 (12)	0.0232 (3)	
H4A	0.5531	0.7191	0.4376	0.028*	
H4B	0.3097	0.6886	0.4182	0.028*	
C5	0.4895 (2)	0.87723 (9)	0.41791 (12)	0.0271 (3)	
H5A	0.4544	0.927	0.3678	0.033*	
H5B	0.6394	0.8619	0.4096	0.033*	
C6	0.1277 (2)	0.83150 (9)	0.38914 (12)	0.0266 (3)	
H6A	0.0318	0.7851	0.3616	0.032*	
H6B	0.087	0.8805	0.3384	0.032*	
N1	0.37969 (18)	0.75391 (7)	0.57675 (10)	0.0208 (3)	
H1	0.409 (3)	0.7097 (12)	0.6156 (16)	0.031*	
N2	0.46828 (19)	0.89757 (7)	0.54400 (10)	0.0243 (3)	
N3	0.09872 (18)	0.85121 (7)	0.51461 (10)	0.0244 (3)	
N4	0.34884 (19)	0.80830 (7)	0.37423 (10)	0.0239 (3)	
C7	0.6729 (2)	0.58907 (8)	0.63638 (11)	0.0176 (3)	
C8	0.7983 (2)	0.50985 (8)	0.64094 (11)	0.0182 (3)	
H8	0.7237	0.4585	0.6406	0.022*	
C9	1.00774 (19)	0.50863 (8)	0.64536 (11)	0.0173 (3)	
H9	1.0825	0.5599	0.6451	0.021*	
C10	1.1315 (2)	0.42962 (8)	0.65074 (11)	0.0180 (3)	
O1	0.46931 (14)	0.58179 (6)	0.62743 (8)	0.0214 (2)	
O2	0.76697 (15)	0.65634 (6)	0.64178 (10)	0.0281 (3)	
O3	1.33649 (14)	0.43696 (6)	0.64341 (9)	0.0220 (2)	
H3	1.382 (3)	0.4983 (12)	0.6347 (15)	0.033*	
O4	1.04798 (15)	0.36217 (6)	0.66187 (10)	0.0305 (3)	

### Atomic displacement parameters (Å^2^)

**Table:**

	*U*^11^	*U*^22^	*U*^33^	*U*^12^	*U*^13^	*U*^23^
C1	0.0283 (7)	0.0205 (7)	0.0229 (6)	−0.0018 (5)	−0.0083 (5)	−0.0022 (5)
C2	0.0381 (8)	0.0157 (6)	0.0269 (7)	0.0061 (5)	−0.0004 (6)	−0.0016 (5)
C3	0.0258 (7)	0.0238 (7)	0.0294 (7)	0.0016 (5)	0.0045 (6)	0.0065 (5)
C4	0.0275 (7)	0.0174 (6)	0.0238 (7)	0.0009 (5)	−0.0016 (5)	−0.0051 (5)
C5	0.0326 (7)	0.0241 (7)	0.0246 (7)	−0.0079 (6)	0.0028 (6)	0.0021 (5)
C6	0.0297 (7)	0.0230 (7)	0.0245 (7)	0.0008 (5)	−0.0093 (6)	0.0024 (5)
N1	0.0259 (6)	0.0135 (5)	0.0216 (5)	0.0013 (4)	−0.0040 (4)	0.0035 (4)
N2	0.0315 (6)	0.0164 (5)	0.0236 (6)	−0.0032 (4)	−0.0034 (5)	−0.0009 (4)
N3	0.0254 (6)	0.0204 (6)	0.0267 (6)	0.0041 (4)	−0.0007 (5)	0.0032 (4)
N4	0.0308 (6)	0.0209 (6)	0.0191 (5)	−0.0019 (5)	−0.0022 (4)	−0.0016 (4)
C7	0.0189 (6)	0.0172 (6)	0.0160 (6)	0.0009 (5)	−0.0015 (4)	0.0007 (4)
C8	0.0200 (6)	0.0152 (6)	0.0185 (6)	−0.0005 (5)	−0.0028 (5)	0.0013 (4)
C9	0.0195 (6)	0.0144 (6)	0.0173 (5)	−0.0013 (4)	−0.0021 (4)	0.0004 (4)
C10	0.0188 (6)	0.0165 (6)	0.0175 (6)	−0.0002 (5)	−0.0037 (4)	0.0005 (4)
O1	0.0167 (4)	0.0185 (5)	0.0284 (5)	0.0017 (3)	0.0000 (4)	0.0021 (4)
O2	0.0241 (5)	0.0155 (5)	0.0428 (6)	−0.0015 (4)	−0.0049 (4)	0.0005 (4)
O3	0.0168 (4)	0.0175 (5)	0.0307 (5)	0.0012 (3)	−0.0020 (4)	0.0028 (4)
O4	0.0253 (5)	0.0167 (5)	0.0484 (7)	−0.0031 (4)	−0.0018 (4)	0.0038 (4)

### Geometric parameters (Å, °)

**Table:**

C1—N2	1.4442 (17)	C5—H5A	0.99
C1—N1	1.5139 (17)	C5—H5B	0.99
C1—H1A	0.99	C6—N4	1.4691 (19)
C1—H1B	0.99	C6—N3	1.4727 (18)
C2—N2	1.4654 (19)	C6—H6A	0.99
C2—N3	1.4695 (18)	C6—H6B	0.99
C2—H2A	0.99	N1—H1	0.845 (19)
C2—H2B	0.99	C7—O2	1.2320 (15)
C3—N3	1.4435 (17)	C7—O1	1.2821 (15)
C3—N1	1.5105 (17)	C7—C8	1.4971 (17)
C3—H3A	0.99	C8—C9	1.3161 (17)
C3—H3B	0.99	C8—H8	0.95
C4—N4	1.4451 (17)	C9—C10	1.4889 (17)
C4—N1	1.5179 (17)	C9—H9	0.95
C4—H4A	0.99	C10—O4	1.2179 (16)
C4—H4B	0.99	C10—O3	1.3081 (15)
C5—N4	1.4715 (18)	O3—H3	1.034 (18)
C5—N2	1.4717 (18)		
			
N2—C1—N1	109.36 (10)	N3—C6—H6A	109.2
N2—C1—H1A	109.8	N4—C6—H6B	109.2
N1—C1—H1A	109.8	N3—C6—H6B	109.2
N2—C1—H1B	109.8	H6A—C6—H6B	107.9
N1—C1—H1B	109.8	C3—N1—C1	109.07 (11)
H1A—C1—H1B	108.3	C3—N1—C4	108.88 (10)
N2—C2—N3	112.14 (11)	C1—N1—C4	108.65 (10)
N2—C2—H2A	109.2	C3—N1—H1	109.9 (12)
N3—C2—H2A	109.2	C1—N1—H1	113.1 (12)
N2—C2—H2B	109.2	C4—N1—H1	107.1 (12)
N3—C2—H2B	109.2	C1—N2—C2	109.24 (11)
H2A—C2—H2B	107.9	C1—N2—C5	109.06 (11)
N3—C3—N1	109.42 (11)	C2—N2—C5	108.26 (11)
N3—C3—H3A	109.8	C3—N3—C2	109.00 (11)
N1—C3—H3A	109.8	C3—N3—C6	109.07 (11)
N3—C3—H3B	109.8	C2—N3—C6	108.38 (11)
N1—C3—H3B	109.8	C4—N4—C6	108.59 (11)
H3A—C3—H3B	108.2	C4—N4—C5	108.84 (10)
N4—C4—N1	109.76 (10)	C6—N4—C5	108.47 (11)
N4—C4—H4A	109.7	O2—C7—O1	123.81 (12)
N1—C4—H4A	109.7	O2—C7—C8	119.76 (11)
N4—C4—H4B	109.7	O1—C7—C8	116.43 (11)
N1—C4—H4B	109.7	C9—C8—C7	122.51 (12)
H4A—C4—H4B	108.2	C9—C8—H8	118.7
N4—C5—N2	112.09 (11)	C7—C8—H8	118.7
N4—C5—H5A	109.2	C8—C9—C10	122.25 (12)
N2—C5—H5A	109.2	C8—C9—H9	118.9
N4—C5—H5B	109.2	C10—C9—H9	118.9
N2—C5—H5B	109.2	O4—C10—O3	121.80 (11)
H5A—C5—H5B	107.9	O4—C10—C9	122.28 (12)
N4—C6—N3	112.10 (11)	O3—C10—C9	115.92 (11)
N4—C6—H6A	109.2	C10—O3—H3	112.0 (9)
			
N3—C3—N1—C1	59.36 (14)	N2—C2—N3—C6	−58.33 (14)
N3—C3—N1—C4	−59.05 (14)	N4—C6—N3—C3	−60.71 (15)
N2—C1—N1—C3	−59.07 (14)	N4—C6—N3—C2	57.83 (14)
N2—C1—N1—C4	59.49 (14)	N1—C4—N4—C6	−59.03 (13)
N4—C4—N1—C3	59.28 (13)	N1—C4—N4—C5	58.87 (14)
N4—C4—N1—C1	−59.39 (13)	N3—C6—N4—C4	60.45 (14)
N1—C1—N2—C2	58.69 (14)	N3—C6—N4—C5	−57.68 (14)
N1—C1—N2—C5	−59.46 (14)	N2—C5—N4—C4	−60.05 (15)
N3—C2—N2—C1	−60.15 (14)	N2—C5—N4—C6	57.93 (14)
N3—C2—N2—C5	58.50 (14)	O2—C7—C8—C9	−3.04 (19)
N4—C5—N2—C1	60.51 (15)	O1—C7—C8—C9	177.28 (11)
N4—C5—N2—C2	−58.25 (15)	C7—C8—C9—C10	179.56 (11)
N1—C3—N3—C2	−59.05 (14)	C8—C9—C10—O4	−6.5 (2)
N1—C3—N3—C6	59.11 (14)	C8—C9—C10—O3	173.79 (11)
N2—C2—N3—C3	60.25 (14)		

### Hydrogen-bond geometry (Å, °)

**Table:**

*D*—H···*A*	*D*—H	H···*A*	*D*···*A*	*D*—H···*A*
N1—H1···O1	0.845 (19)	2.094 (19)	2.8704 (14)	153 (2)
O3—H3···O1^i^	1.034 (18)	1.458 (18)	2.4877 (13)	174 (2)
C1—H1A···O3^ii^	0.99	2.46	3.2708 (16)	138
C3—H3B···O4^iii^	0.99	2.55	3.4629 (18)	154
C4—H4B···O4^iv^	0.99	2.48	3.3668 (17)	149
C6—H6A···O4^iv^	0.99	2.43	3.3352 (18)	152

Symmetry codes: (i) *x*+1, *y*, *z*; (ii) −*x*+2, *y*+1/2, −*z*+3/2; (iii) −*x*+1, *y*+1/2, −*z*+3/2; (iv) −*x*+1, −*y*+1, −*z*+1.
